# Portal Vein Tumor Thrombus: No Longer a Death Sentence

**DOI:** 10.7759/cureus.12845

**Published:** 2021-01-21

**Authors:** Phillip Esposito, Mika Matteo, Carissa Concepcion, Matthew Montanarella, Jerry Matteo

**Affiliations:** 1 Radiology, University of Florida College of Medicine, Jacksonville, USA

**Keywords:** cryoablation, macrovascular invasion portal vein thrombus, hepatocellular carcinoma, minimally invasive treatment, palliative cryoablation

## Abstract

Hepatocellular carcinoma (HCC) is the most common malignancy of the liver and a leading cause of cancer mortality worldwide. HCC commonly results from longstanding liver cirrhosis, which presents a host of complications and a severely diminished quality of life. Despite advancements in diagnosis, molecular pathogenesis, and management of the complications associated with irreversible liver diseases, HCC remains an aggressive malignancy with high mortality. HCC is often invasive to adjacent vasculature, including the inferior vena cava (IVC) and portal veins, which present with rapid morbidity and patient decline. This article describes a patient with cirrhosis and HCC previously treated with cryoablation now presenting with multiple new foci and invasion of the left medial portal vein. These lesions were synchronously cryoablated. Cryoablation is typically reserved for solid tumor masses within the soft tissue or specific organs. This report illustrates a technique of directly cryoablating tumors within vessels. We achieved adequate cryoablation of the intravascular HCC portal vein tumor thrombus in the left medial portal vein. A one-month follow-up CT scan demonstrated significant portal vein macrovascular invasion (MVI) regression from 22.8 mm to 7.7 mm. Portal vein invasion by HCC presents unique challenges and traditionally precludes percutaneous or surgical therapy. Our technique shows successful cryoablation of MVI as a viable adjunct to treatment in select patients.

## Introduction

Hepatocellular carcinoma (HCC) is the most common primary liver malignancy and the sixth most common cancer worldwide. Additionally, it is the fourth most common cause of cancer-related mortality worldwide. Annually, HCC is responsible for approximately 782,000 deaths and 841,000 new cases worldwide, and its incidence has increased by 75% between 1990 and 2015 [1-2]. Common causes of HCC, and more broadly, cirrhosis, include chronic hepatitis B and C infections, chronic alcoholism, hemochromatosis, Wilson disease, aflatoxin ingestion, epigenetic inheritance, alpha-1-antitrypsin disease, and non-alcoholic fatty liver disease [3]. A herbal mutagen aristolochic acid, a toxic ingredient of Chinese herbal preparation of wild ginger, has been implicated most recently [4]. Globally, HCC has a ratio of mortality to incidence of 0.95, indicating that obtaining disease-free status is rare, although early staging and therapy may improve mortality [5]. Despite surveillance protocols for patients with cirrhosis and screening guidelines for hepatitis B and C from the United States Preventative Services Task Force (USPSTF), most cases of HCC are diagnosed at intermediate or advanced stages and often involve the vasculature, including the portal vein, hepatic vein, or inferior vena cava.

Late manifestations of HCC include contiguous invasion of adjacent hepatic vessels where the tumor extends into the inferior vena cava (IVC) and portal vein, known as a “tumor thrombus.” This phenomenon of vascular extension and tumor thrombus also occurs in renal cell carcinoma (RCC) [6]. The result is diminished hepatic blood circulation, diminished venous return to the heart, portal hypertension, extensive variceal anastomotic blood flow, variceal hemorrhage, gastrointestinal bleeding, fluid overload and ascites, hepatorenal syndrome, and splenomegaly which contribute to patient demise. The two most critical preoperative factors to predict prognosis are clinically relevant portal hypertension and elevated bilirubin [7].

Treatment options for HCC include liver transplantation, surgical resection, transarterial chemoembolization (TACE), percutaneous ethanol injection, percutaneous radiofrequency ablation, and percutaneous cryoablation [7-8]. Percutaneous therapy is preferred in patients with contraindications to surgical resection as it is minimally invasive and performed under monitored anesthesia care (MAC) and local anesthetic agents.

Percutaneous cryoablation (PC) was first reported in the literature in the late 1990s, where some institutions employed a follow-up treatment with ethanol ablation for incomplete therapy or recurrent disease. However, cryoablation alone is most commonly used today. Cryoablation is a minimally invasive surgical procedure where probes, under computed tomography (CT) guidance, are placed into cancerous tissue and lethal temperature (approaching -140° F) is applied directly to the tumor. Due to the focal freeze zone, only the necessary tissue amount is destroyed, allowing adjacent normal tissue to regenerate and maintain the liver reserve. This is important in HCC, where patients often have cirrhosis and decreased regenerative capacity and functional reserve. PC is optimal for patients with multiple foci of disease in whom transplant or surgical resection is contraindicated as multiple probes can be placed to simultaneously target each lesion [9]. With advanced disease or when vessel invasion is present, effective cryoablation becomes difficult due to a thermal “heat-sink” effect, whereby continuous blood flow within the vessel warms the tissue and prevents optimal freeze temperature [10]. In suboptimal cases, such as extensive vessel invasion, this effect can be significant. Cryoablation within vascular structures has rarely been reported in the literature and generally precludes surgical intervention or cryoablation in most facilities [8]. A technique to treat HCC with an IVC invasion has been described whereby balloon angioplasty was employed to limit blood flow and thawing of the ice ball during the freeze cycles [11]. We describe a technique using cryoablation probes placed directly within the portal vein tumor thrombus.

## Technical report

**Figure 1:**
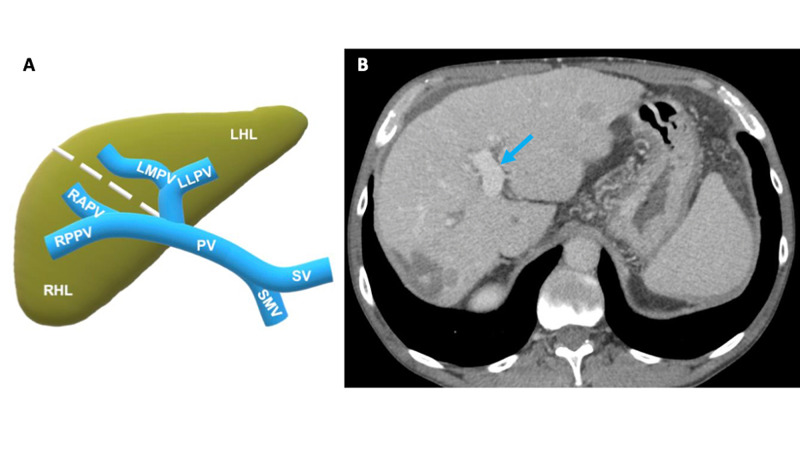
1A: schematic of the left and right hepatic lobes and the portal vein circulation; 1B: contrast-enhanced axial abdominal CT four months prior to the finding of the portal vein tumor thrombosis On this CT, the portal vein was widely patent without evidence of tumor (blue arrow). CT: computed tomography; LHL: left hepatic lobe; LLPV: left lateral portal vein; LMPV: left medial portal vein; PV: portal vein; RAPV: right anterior portal vein; RHL: right hepatic lobe; RPPV: right posterior portal vein; SMV: superior mesenteric vein; SV: splenic vein

The patient was a 61-year-old male with a five-year history of recurrent HCC with multiple previous cryoablations and chemoembolization procedures. The recent cryoablation was performed on liver segments 4A, 4B, and the left medial portal vein due to the patient being a poor surgical candidate due to multifocal disease and large vessel involvement. Five years before this procedure, the patient was first known to have cirrhotic changes with nodules, small volume ascites, coarse echotexture, and progressive nodularity. The patient had an ill-defined 1.7 x 1.5 x 1.4 cm hypoechoic lesion in the left hepatic lobe and a 1.2 x 0.9 x 1.1 cm hypoechoic lesion in the right hepatic lobe noted on screening ultrasound (US). These lesions were redemonstrated as two ill-defined enhancing lesions on CT with signs of portal hypertension and possible satellite lesions. Biochemistry lab results showed elevated alpha-fetoprotein (AFP), and a biopsy confirmed HCC. Magnetic resonance imaging (MRI) with a liver protocol was performed shortly thereafter and redemonstrated lesions compatible with the diagnosis of HCC. The two lesions were treated in the interventional radiology (IR) suite with mitomycin TACE, followed by CT-guided cryoablation two days later. Surveillance CTs over the next four years were routinely noted to have recurrent malignancy, and the patient was treated multiple times with mitomycin TACE, microwave ablation, and cryoablation. Routine surveillance CT four months prior to the macrovascular invasion (MVI) finding shows a widely patent portal vein (Figure 1). 

**Figure 2:**
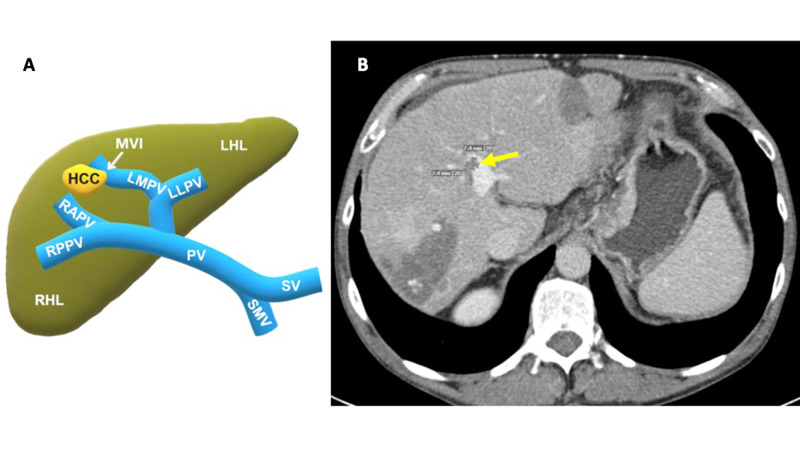
2A: schematic of the new HCC macrovascular invasion of the left medial portal vein; 2B: contrast-enhanced axial abdominal CT, four months after Figure 1B, demonstrating a new 7.4 mm x 7.4 mm invasive macrovascular tumor thrombus within the left medial portal vein (yellow arrow) CT: computed tomography; HCC: hepatocellular carcinoma; LHL: left hepatic lobe; LLPV: left lateral portal vein; LMPV: left medial portal vein; MVI: macrovascular invasion; PV: portal vein; RAPV: right anterior portal vein; RHL: right hepatic lobe; RPPV: right posterior portal vein; SMV: superior mesenteric vein; SV: splenic vein

The decision was made to perform cryoablation on all four liver lesions and the new tumor thrombus within the left medial portal vein (Figure 2). 

**Figure 3:**
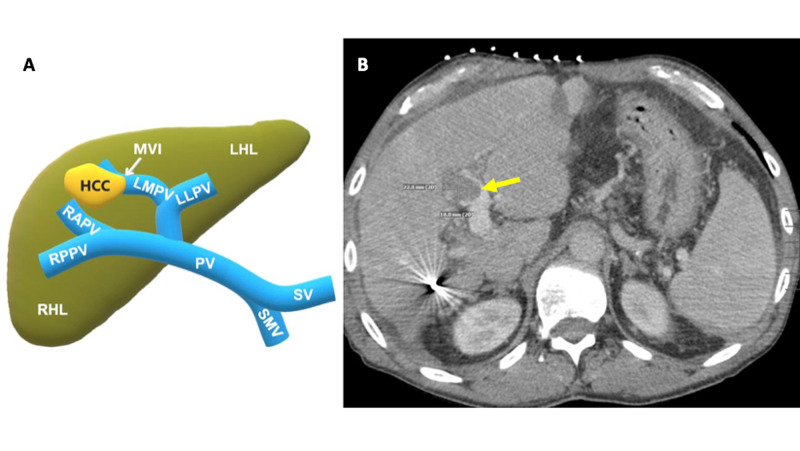
3A: schematic representation of the liver with portal circulation and the rapidly growing aggressive HCC lesion; 3B: contrast-enhanced axial abdominal CT showing an aggressive, fast-growing HCC with macrovascular extension into and adjacent to the left medial portal vein that had significantly grown to 22.8 mm x 18 mm (yellow arrow) CT: computed tomography; HCC: hepatocellular carcinoma; LHL: left hepatic lobe; LLPV: left lateral portal vein; LMPV: left medial portal vein; MVI: macrovascular invasion; PV: portal vein; RAPV: right anterior portal vein; RHL: right hepatic lobe; RPPV: right posterior portal vein; SMV: superior mesenteric vein; SV: splenic vein

Five weeks later, the patient returned for this procedure. The patient had adequate pre- and postoperative cardiorespiratory function and normal routine biochemistry laboratory values. After appropriate informed consent was obtained, the patient was placed supine on the CT table, and the right upper quadrant was prepped and draped in the typical sterile fashion. Monitored anesthetic care with Versed® (midazolam) and fentanyl was utilized for moderate sedation. Limited CT images were obtained to identify the liver masses and for pre-procedure planning. The skin was marked and anesthetized with 2% lidocaine. An intraoperative planning CT was obtained that showed a contrast-enhanced axial abdominal CT with an aggressive, fast-growing tumor that had significantly grown to 22.8 mm x 18 mm (Figure 3). 

**Figure 4:**
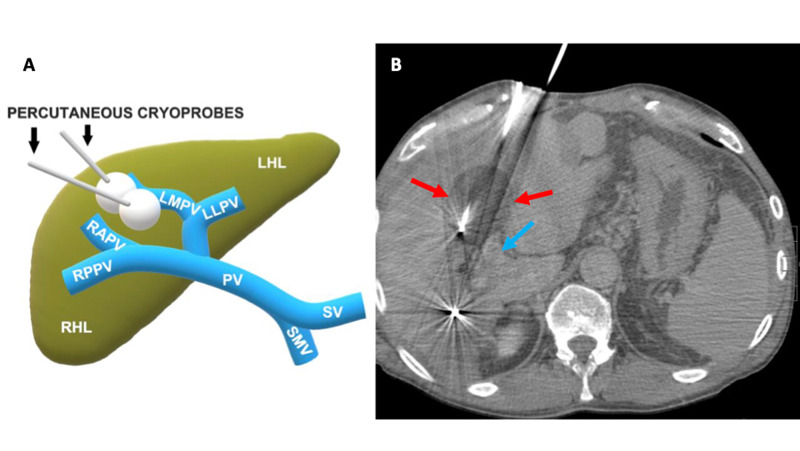
4A: schematic drawing of the liver with portal circulation and two percutaneous cryoprobes with ice balls ablating the invasive HCC; 4B: contrast-enhanced, axial abdominal CT with two adjacent cryoprobes located directly within the tumor thrombus and ice ball formation (red arrows) The blue arrow depicts the left portal vein. CT: computed tomography; HCC: hepatocellular carcinoma; LHL: left hepatic lobe; LLPV: left lateral portal vein; LMPV: left medial portal vein; MVI: macrovascular invasion; PV: portal vein; RAPV: right anterior portal vein; RHL: right hepatic lobe; RPPV: right posterior portal vein; SMV: superior mesenteric vein; SV: splenic vein

Using Boston Scientific’s VISUAL ICE™ Cryoablation System (Boston Scientific Corp., Marlborough, MA, USA), a total of seven IceEdge™ 2.4 Cryoablation Needle probes were inserted into the four separate tumor lesions. Two probes were placed directly into the left medial portal vein tumor thrombus (Figure 4). 

All tumors underwent cryoablation simultaneously using two 10-minute freeze cycles interspersed with two five-minute thawing cycles. Limited CT images were obtained during and after the freeze cycles to ensure adequate ice formation encompassing all masses. This demonstrated sufficient coverage of all four masses. All cryoprobes were removed, and a final postoperative limited CT was performed, revealing appropriate hypodensities within each ice ball's regions. The left portal vein was patent. Sterile dressings were applied, and the patient was sent to the recovery holding bay and discharged home the same day.

**Figure 5:**
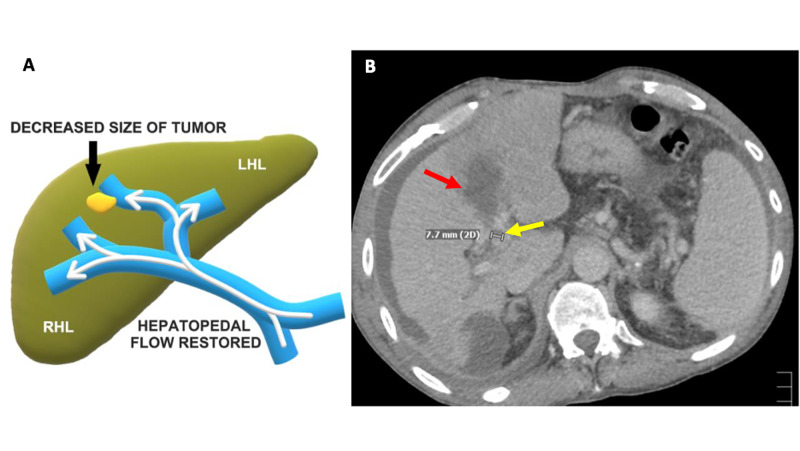
5A: schematic representation of the HCC lesion at the one-month follow-up demonstrating significant shrinkage; 5B: one-month follow-up contrast-enhanced axial abdominal CT demonstrating a decreased size of the intravascular left medial portal vein tumor thrombus measuring 7.7 mm (yellow arrow) and post-ablation nonviable tissue at the cryoablation site (red arrow) CT: computed tomography; HCC: hepatocellular carcinoma; LHL; left hepatic lobe; RHL; right hepatic lobe

The patient tolerated the procedure well without any intraoperative complications. The patient returned two weeks later for follow-up which demonstrated an increase in intraabdominal ascites that was subsequently removed with therapeutic paracentesis. The patient had a transient postsurgical increase in liver enzymes following the procedure which quickly returned to baseline. Follow-up imaging one month after the procedure demonstrated the ablated zones with nonviable tissue and considerable reduction in tumor burden of the left medial portal vein. The lesion then measured 7.7 mm reduced from 22.8 mm a month prior (Figure 5).

## Discussion

HCC may invade major vessels through stromal and endothelial destruction, which may cause flow-limiting stenosis or thrombus formation. Life expectancy with portal vein tumor thrombosis is two to four months with only supportive care [12]. Tumor cells can proliferate within the vessel lumen. They can either serve as a nidus for thrombus formation or become coated with endothelium, protecting it from activating the coagulation cascade. In the latter case, there appears to be an increased propensity of distant metastasis of endothelial-coated microthrombi, especially to the pulmonary circuit [11, 13]. These microthrombi may retain viability at distant capillary beds and within the adjacent hepatic circulation, thus providing a mechanism for distant metastasis [14].

Endothelial-coated HCC is, in part, cloaked from the immunosurveillance mechanism, preventing neoantigen recognition, complement-mediated destruction, and clotting factor activation [11]. Additionally, endothelial-coated HCC appears to augment vascular growth factors by expressing angiopoietin-2, an essential mediator for the activation of the vascular endothelial growth factor (VEGF) pathway angiogenesis [14]. 

Standard of care guidelines for HCC is recommended by the European Association for the Study of the Liver (EASL) using the Barcelona-Clinic Liver Cancer (BCLC) classification dividing HCC patients into five stages (0, A, B, C, and D). Portal involvement is automatically designated as an advanced stage “C”. Sorafenib, a VEGF inhibitor, is currently the only therapy option [3]. An article by Rong et al., reviewing 1,197 HCC lesions treated with cryoablation, was noted to have a complete response to therapy in 1,163 (96.1%) lesions with an overall complication rate of just 2.8%, with no treatment-related mortalities [15]. Knowing the dismal prognosis of HCC with MVI, our procedure offered the patient an improved quality of life and decreased morbidity. A literature review shows only a single article describing cryoablation of a macrovascular HCC tumor thrombus [11]. The Soule and Matteo article describes a patient with HCC and invasion to the IVC who successfully underwent percutaneous cryoablation therapy of an MVI tumor and showed a significant reduction in morbidity and mortality [11].

This intravascular cryoablation technique has a 100% success rate at our institution with this described patient and an additional patient with hepatocellular carcinoma with IVC invasion described in the article by Soule and Matteo [11]. To our knowledge, this technique has not been performed at any other institution or published in the literature and may add another palliative treatment option for patients with end-stage hepatocellular carcinoma. Additional studies demonstrating long-term outcomes of this novel technique may prove it to be a viable standard treatment option for HCC with macrovascular invasion. A limitation of this report was the inherent inability to use a balloon within the portal vein to temporarily halt blood flow at the site of cryoablation with a resultant thermal “heat-sink” effect. The technique was successful despite this inherent limitation. 

## Conclusions

Hepatocellular carcinoma with macrovascular invasion poses a grim prognosis for patients with few opportunities for palliative treatment. Cryoablation has been successful in the palliative debulking of soft tissue tumors of the gastrointestinal tract, and this technique can be used to treat tumors with spread into neighboring vessels. This technique was successful in shrinking the size of the tumor while preserving viable hepatic tissue and portal vein patency. This article creates a model to pursue further research of cryoablation of macrovascular invasive HCC with portal vein tumor thrombus in select nonsurgical candidates with preserved liver function who statistically have months to live. 
